# Systemic immunosuppressive therapy in juvenile idiopathic arthritis-associated uveitis

**DOI:** 10.1007/s10792-026-04056-0

**Published:** 2026-04-02

**Authors:** Mine Esen Baris, Deniz Bagci, Halil Ates, Suzan Guven

**Affiliations:** Department of Ophthalmology, Ege University, Izmir, Turkey

**Keywords:** Anterior uveitis, Biological agents, Juvenile idiopathic arthritis, Immunosuppressive treatment

## Introduction

Juvenile idiopathic arthritis (JIA) is a type of idiopathic, chronic arthritis that presents before the age of 16 and lasts for more than 6 weeks [1, 2]. It has an estimated prevalence of 7–21 cases per 100,000 individuals in the United States (US) and Northern Europe [3, 4]. It can have a negative impact on the growth and development of the skeletal system [3, 5, 6]. But it also constitutes a significant risk of severe ocular damage and blindness, via anterior uveitis. Uveitis has been reported in 10–20% of patients with juvenile idiopathic arthritis, but due to the silent inflammation, the real numbers might be higher [7]. Girls with oligoarthritis who are younger than seven years old and have a positive antinuclear antibody (ANA) test are especially under a higher risk of developing JIA-associated uveitis [7, 8].

The standard initiation theraphy for JIA-associated uveitis is topical and systemic corticosteroids, typically supplemented with disease-modifying anti-rheumatic drugs (DMARDs) such as methotrexate. The significant role of tumor necrosis factor-alpha (TNF-α) in the development of uveitis has been demonstrated in previous studies and in cases where standard treatment and second-line DMARD therapy fail, anti-TNF agents have been used successfully for treatment in non-infectious uveitis, including the ones associated with JIA [9–11].

Two placebo-controlled, double-blind, randomized studies, the SYCAMORE and the ADJUVITE studies have demonstrated anti-TNF antibody adalimumab is effective in patients who are resistant or have inadequate response to local steroid therapy and methotrexate (MTX) [12, 13]. Intravenous infliximab and subcutaneous golimumab are reported to be effective, but there are no randomized controlled trials [14, 15].

The purpose of this study is to evaluate the long term treatment results of systemic immunosupresive treatment and analyze the effects of biologic agents on corticosteroid exposure and clinical findings in JIA associated uveitis.

In our clinic, the treatment protocol of pediatric chronic noninfectious uveitis includes conventional immunsupresive treatment for all patients unless contraindicated. As the initial step, MTX was started at a dose of 15 mg/m^2^/week, and in severe cases, systemic corticosteroid therapy was initiated at a dose of 1–2 mg/kg/day, tapered down to < 10 mg/day for maintenance. Despite the initial treatment mentioned, patients with persistant/recurrent anterior segment inflammation (presence of cells or increased flare in anterior chamber despite MTX and maintenance dose of corticostroids) required anti-TNFα agents and adalimumab was the treatment of choice unless contra-indicated or unless other anti-TNFα agents were a necessity for extra-ocular findings. During acute anterior uveitis relaps periods, all patients used topical corticosteroids (1% prednisolone) and cycloplegic agents.

The efficacy of anti-TNF-alpha on uveitis activity was assessed based on clinical examination findings (inflammatory cells in anterior chamber and aqueous flare were graded according to Standardization of Uveitis Nomenclature (SUN) criteria [15].

## Materials and methods

The files of patients followed with the diagnosis of JIA-associated uveitis between 2007 and 2022 in our clinic were retrospectively reviewed. The demographic characteristics of all patients (Table 1), clinical findings including best corrected visual acuity (BCVA), intraocular pressure (IOP), biomicroscobic anterior segment findings and posterior segment examination findings with 90 D lens at first visit, 1st, and 2nd year visits, in addition to 5th year visit were recorded. Flare values measured by laser flaremeter (Kowa FM 600, Hamamatsu, Japan) number of uveitis relapses per year (identified as cells or flaremeter results of more than 7.5 photons/msec in aqueous humor), treatment protocols (including the initiation and discontinuation dates as well as reasons for change in treatment), and medication side effects were analyzed. Number of pre-treatment uveitis relapses were recorded at first visit, based on family’s statement. During relapses, all patients were treated with topical corticosteroids and cycloplegic agents. Systemic corticosteroids were added for patients who did not respond to topical therapy. All patients received methotrexate (MTX) treatment. Anti-TNFα agents were required in patients due to persistent anterior segment inflammation despite weekly systemic MTX therapy. Anti-TNFα treatment was discontinued after 2 years of no relapses.

**Table 1:** Demographic data of all participants

Number of patients, n (%)	39(100)
Number of eyes, n(%)	78 (100)
Gender, n(%)	
Female	18 (46.1)
Male	21 (53.8)
Age at diagnosis, years, mean ± SD (min.-max.)	8.6 ± 4.2 (2–18)
Duration of follow-up, years, mean ± SD (min.-max.)	6.8 ± 3.7 (1–15)
Age at initiation of Anti-TNFα, years, mean ± SD (min–max)	11.0 ± 3.8 (5–20)
Duration of Anti-TNFα use, months, mean ± SD (min–max)	24.4 ± 17.1 (6–68)

Children under 18 years of age who were regularly attending follow-ups (which were scheduled in every 2 months unless there are any symptoms of active inflammation) and had been confirmed with JIA diagnosis by peadiatric rheumatology department were included in the study. Patients who did not adhere to treatment and those with non-JIA childhood uveitis were excluded from the study. Treatment non-adherence was defined as not using the prescribed medications or taking medications other than the ones that were prescribed by the study center and/or not attending follow up visits according to the suggested timeline. Patients who were followed up for less than 12 months were also excluded.

For analysis, The Statistical Package for Social Sciences (SPSS) program (IBM SPSS Statistics for Windows, Version 25.0. Armonk, NY: IBM Corp.) was used. Descriptive statistics were given as mean, standart deviation, median, minimum, maximum frequency, and percentage values. The Shapiro–Wilk test was used to test the normality assumptions of the quantitative data. Dependent samples *t* test was used for normally distributed variables. The statistical significance value was defined as *p* < 0.05.

## Results

The mean age at diagnosis for the 39 (18 F, 21 M) patients included in the study was 8.6 ± 4.2 (3–18) years, with a mean follow-up duration of 6.8 ± 3.7 (1–15) years. All patients had bilateral uveitis. Sixteen patients were excluded due to treatment non-adherence and 3 patients were excluded due to short follow-up. While all cases had anterior uveitis, anterior vitreous cells and inflammation were detected in 18 (46.1%) cases in addition to inflammatory cells in anterior chamber. The mean BCVA-LogMAR at the initial visit showed a significant increase from 0.15 ± 0.52 (3.1–0) to 0.1 ± 0.6 (2–0) at the 5th year visit (*p* = 0.018) (Table 2). The mean number of uveitis relapses decreased from 2.9 ± 1.7 relapses/year at the initial visit to 0.8 ± 1.1 relapses/year at the 5th year visit (*p* < 0.001).

**Table 2:** Clinical findings of all study eyes before and after systemic immunosuppressive treatment

	Before treatment	5th years	*P* value
BCVA- LogMAR, mean ± SD (min–max)	0.15 ± 0.52 (3.1–0)	0.1 ± 0.6 (2–0)	0.018
"Mean ± SD (min–max) relapses/year"	2.9 ± 1.7 (0–7)	0.8 ± 1.1 (0–4)	0.001
İntraocular pressure (IOP), mean ± SD (min- max) (mmHg)	15.1 ± 6.5 (6–35)	13.8 ± 5.1 (7–42)	0.09
Cup-to-disc (C/D) ratio mean ± SD (min–max)	0.25 ± 0.14 (0.1–1)	0.28 ± 0.21 (0.1–1)	0.6

Number of patients: 39

BCVA, Best corrected visual acuity; Min, minimum; Max, maximum; JIA, Juvenile idiopathic arthritis

During relapses, all patients were treated with topical corticosteroids and cycloplegic agents. Systemic corticosteroids (prednisolone acetate 1 mg/kg/day) were added in 11 patients (30.5%) who did not respond to topical therapy. All patients received MTX (15 mg/m2/week) treatment (no contrindication was present). The follow-up period before the start of i immunosuppressive treatment was 4.14 ± 1.95 (2–8) weeks. Before starting immunosuppressive therapy, patients received only topical treatment. Anti-TNFα agents were required in 21 (53.8%) patients due to persistant anterior segment inflammation despite weekly systemic immunosuppressive treatment.

**Fig. 1:**
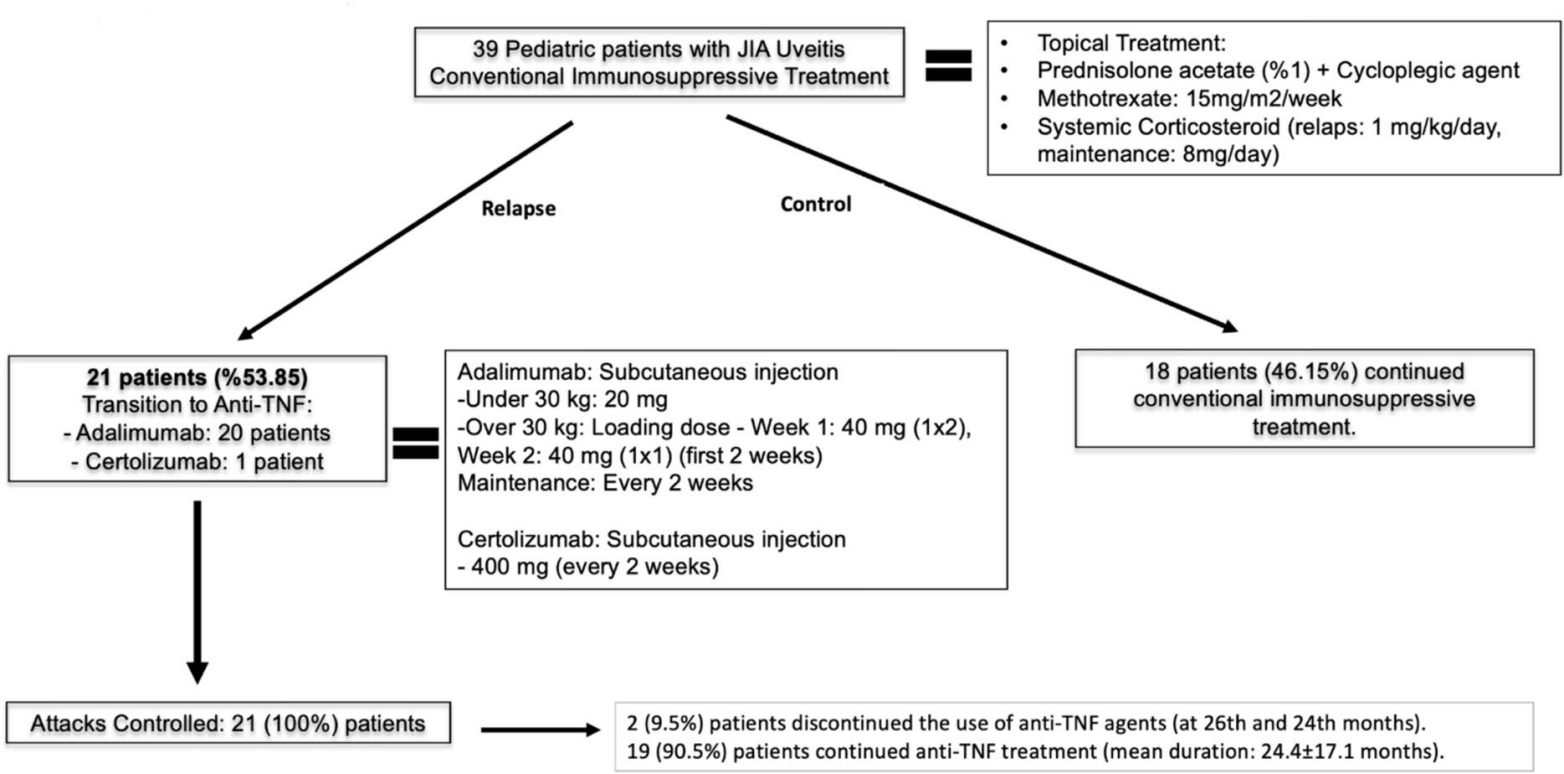
Treatment protocols of patients

Among the 21 patients receiving anti-TNFα therapy, 20 (95.2%) were on adalimumab, and 1 (4.8%) was on certolizumab, which was initiated as per the recommendations of pediatric rheumatology department (Fig. 1).

The mean age at initiation of anti-TNFα therapy was 11.0 ± 3.8 (5–20) years, with a mean duration of medication use of 24.4 ± 17.1 (6–68) months. A significant increase in mean BCVA (from 0.7 ± 0.4(3.1–0) to 1 ± 0.3 (1.8–0) LogMAR) and a significant decrease in uveitis relaps frequency (from 3 ± 1.6 to 0.9 ± 1.1 relapses/year) were observed after 2 years of anti-TNFα therapy (both *p* < 0.001) (Table 2, 3). Among patients receiving anti-TNFα therapy, 10 (47.6%) patients did not require any additional systemic corticosteroids, and no serious complications of the anti-TNFα drug were encountered. Anti-TNFα agents could be discontinued in 2 patients (9.5%) who had no relapses during 2 years (one in the 26th month and the other in the 24th month of treatment).

**Table 3:** Clinical findings, mean systemic and topical corticosteroid doses before and after the initiation of anti-TNFα treatment

	Before treatment_1_	First year of anti-TNFα treatment_2_	Second year of Anti-TNFα treatment_3_	P_1-2_	P_1-3_	P_2-3_
BCVA- Snellen chart, mean ± SD (min- max)	0.7 ± 0.4 (3.1–0)	0.1 ± 0.7 (1.3–0)	1 ± 0.3 (1.8–0)	0.001	0.001	1.2
Relapses/year Mean ± SD (min–max)	3.0 ± 1.6(1–7)	1.1 ± 0.8 (0–5)	0.9 ± 1.1 (0–4)	0.002	0.001	0.07
Intraocular pressure (IOP), mean ± SD (min–max) (mmHg)	19.8 ± 5.4 (13.1–25.3)	12.7 ± 4.6 (9.6–20.8)	15.3 ± 3.4 (10.2–21.5)	0.02	0.04	0.08
Cup-to-disc (C/D) ratio, mean ± SD (min–max)	0.28 ± 0.18 (0.1–1)	0.3 ± 0.2 (0.1–1)	0.3 ± 0.21 (0.1–1)	0.8	0.8	1.2
Systemic corticosteroid dose, mean ± SD (min.-max.), mg/day	10,7 ± 9.2 (0–32)	2.4 ± 1.3 (0–8)	1.5 ± 1.4 (0–8)	0.001	0.001	0.08
Topical corticosteroid dose, mean ± SD (min.-max.), drops/day	4.8 ± 1.4 (2–6)	1.1 ± 0.9 (0–3)	1.1 ± 0.7 (0–6)	0.003	0.003	0.9

Number of patients: 21

BCVA, Best corrected visual acuity; SD, standard deviation; Min, minimum; Max, maximum

The mean systemic corticosteroid dose was 6.36–8.3(0–64) mg/day before anti-TNFα therapy, and decreased to 2.51–8.72(0–64) mg/day in the 2nd year anti-TNFα therapy. (*p*:0.02).

At the time of diagnosis, the mean cup-to-disc (C/D) ratio of 39 patients with JIA-U was 0.25 ± 0.14 (0.1–1), and the mean intraocular pressure (IOP) was 15.17 ± 6.55 (6–35) mmHg. At the fifth-year visit, the mean C/D ratio was 0.28 ± 0.21 (0.1–1) (*p* > 0.05), and the mean IOP was 13.81 ± 5.12 mmHg (7–42) (*p* < 0.05). The mean pre-treatment IOP of patients scheduled for anti-TNFα therapy was 19.8 ± 5.4 (13.1–25.3) mmHg, which decreased to 14.7 ± 3.5 (9.9–21.5) mmHg (*p* = 0.04) after 2 years of treatment. The mean pre-treatment cup-to-disc (C/D) ratio of patients scheduled for anti-TNFα therapy was 0.28 ± 0.18 (0.1–1) and remained stable at 0.3 ± 0.21 (0.1–1) (*p* > 0.05) following 2 years of treatment.

The most common ophthalmological complications observed in eyes with uveitis were cataracts in 18 (42.9%) eyes, glaucoma in 11 (26.2%) eyes, band keratopathy in 10 (23.8%) eyes, and cystoid macular edema in 4 (2.3%) eyes (Table 4). No patient had cataract surgery. The mean time from JIA-U diagnosis to glaucoma development was 19.33 ± 32.6 months (3–121). Among the 11 eyes with glaucoma, topical antiglaucoma agents were sufficient to lower IOP in 5 eyes, whereas glaucoma surgery was performed in 6 eyes (Table 5).

**Table 4:** The most common ophthalmological complications

Complication	Number of eyes
Cataract, n (%)	18 (23.0)
Glaucoma, n (%)	11 (14.1)
Band keratopathy, n (%)	10 (12.8)
Cystoid macular edema, n (%)	4 (5.1)

**Table 5:** Patients whose intraocular pressure was controlled with glaucoma surgery

Glaucoma surgery, n (%)	6 (14.2)
Trabeculectomy	3 (7.14)
Deep sclerectomy	2 (4,7)
Glaucoma drainage device	1 (2.3)

## Discussion

Juvenile idiopathic arthritis-associated uveitis (JIA-U) remains a significant cause of visual morbidity in pediatric patients. In this study, a significant improvement in BCVA and reduction in uveitis relapses per year were observed after the initiation of systemic immunosuppressive treatment with methothrexate and anti-TNFα therapy in eyes with JIA-U. But as common knowledge, it’s not always possible to control the severe inflammation with conventional immunosuppressives alone and systemic and topical corticosteroids are commonly applied. As a result of the intense research to control the severe inflammation and reducing the corticosteroid exposure, biological agents, are now an essential part of the treatment of chronic non-infectious uveitis, in both children and adults [16]. A study from the UK reported that 47.9% of the children with non-infectious required additional biological therapy in addition to MTX [17–19, 19]. In our study, 53.8% of the eyes with JIA-U required biological agents. Initiating anti-TNFα treatment in these patients significantly decreased the number of uveitis relapses per year, increased the BCVA and reduced systemic and topical corticosteroid exposure in both first and second year of the treatment compared to pre- anti-TNFα period (*p* = 0.001 and *p* = 0.003 respectively for systemic and topical exposure). These results suggest that early and sustained intervention with biologics can preserve and even improve visual function. The significant reduction in IOP is thought to be partly due to the medical and/or surgical glaucoma treatment applied to 11 eyes with glaucoma (nearly half of the eyes receiving anti-TNFα therapy) or the decrease in corticosteroid exposure, which may have contributed to the reduction in IOP.

In a study by ElMohsen et al. [20], the percentage of patients who successfully reduced steroid use with biological therapy ranged from 31.4 to 66.7%. When considering only systemic steroid therapy, this rate increased to between 71.4 and 88.6%, showing a rising trend with longer follow-up periods. Similarly, Vazquez-Cobian et al. [21] found that adalimumab reduced the need for topical corticosteroid eye drops in 78.5% of their patients. In 2007, Biester et al. [22] demonstrated that adalimumab was effective in 83% of patients when systemic steroid use was discontinued. The SYCAMORE study, which compared JIA patients receiving MTX and adalimumab to those receiving MTX and a placebo, reported similar findings [12]. The authors concluded that, compared to the placebo group, patients in the adalimumab group were more likely to require either a dose reduction (*P* = 0.04) or complete cessation of topical glucocorticoid therapy (*P* = 0.02) [12]. In a study by ElMohsen et al. [20], 88.86% of patients were using MTX. In patients receiving concomitant immunomodulatory drugs, significant success in reducing steroid use (at 12 and 24 months of follow- up) and complete success (at 12 months) were found compared to those not receiving concomitant immunomodulatory drugs. In our study, in addition to significantly reducing the corticosteroid exposure, more than half of the patients with anti-TNFα treatment did not require systemic corticosteroid treatment again, during the follow up period.

The chronic and silent nature of the disease and inadequate control of the inflammation often leads to sequelae causing visual impairment. [23] Combination of adalimumab and MTX is reported to be more effective than MTX alone [24]. Also it has been reported that combining MTX with anti-TNFα agents increases tolerance to possible antibody reactions caused by adalimumab or infliximab therapy [25, 26]. In our study, a significant improvement in mean visual acuity and a reduction in the annual relapse rate were observed both after the initiation of MTX and following the start of anti-TNFα therapy. Similar to our finding of decreased number of uveitis relapses (from 3.0 ± 1.6 to 0.9 ± 1.1 relapses/year) after anti-TNFα therapy, Choe et al. studied 22 eyes treated with adalimumab for 12 months and observed a significant decrease in number of uveitis relapses.

Infliximab and adalimumab are the primary anti-TNFα agents used for treating refractory or chronic childhood uveitis, whereas etanercept is less frequently prescribed, particularly after being associated with 43 cases of uveitis in a registry-based study (27). Other studies have also supported this association [27, 28]. Currently, adalimumab—the first FDA-approved biological agent prescribed at our hospital—is administered subcutaneously. Golimumab and certolizumab, which have demonstrated efficacy in rheumatic diseases, are potential future alternatives for JIA-associated uveitis [29]. A meta-analysis by Yulu Li et al. [30] indicated that adalimumab is more effective and has fewer side effects than infliximab, while infliximab is more effective than etanercept. However, there is limited research on the efficacy of golimumab and certolizumab. In a multicenter study by Martín-Varillas JL et al. [31], which included patients with a mean age of 41.6 ± 11.7 years, certolizumab was found to be both effective and safe for Caucasian patients with immune-mediated inflammatory diseases and resistant uveitis in both the short and long term. In our study, the patient who received certolizumab was initiated on treatment by the rheumatology department.

While controlling inflammation is essential to prevent complications, studies have shown that 60% of eyes experience an increase in IOP even when ocular inflammation is inactive [32]. Skarin et al. [33] analyzed a cohort of 55 JIA-U patients between 1973 and 1982, reporting that after 7 years, 42% of eyes had cataracts and 5% had glaucoma. After 24 years, these rates increased to 51% and 22%, respectively. Notably, 49% of eyes still showed signs of active uveitis or were under treatment for recent disease activity [33]. In this study, glaucoma was observed in 11 eyes (26.2%) after a mean of 19.33 ± 32.6 months (3–121). Among the 11 eyes with glaucoma, topical antiglaucoma agents were sufficient to lower IOP in 5 eyes, while glaucoma surgery was performed in 6 eyes. Regular intraocular pressure (IOP) monitoring at each visit is crucial for these patients due to the high risk of developing uveitic glaucoma or ocular hypertension.

## Conclusion

The main disadvantage of our study was its retrospective design and lack of a control group. But longer follow up period provided some valuable information. The most common ophthalmological complications in our study were cataracts in 18 (42.9%) eyes, glaucoma in 11 (26.2%) eyes, band keratopathy in 10 (23.8%) eyes, and cystoid macular edema in 4 (2.3%) eyes. All these vision threatening complications are either a direct result of inflammation or a result of the corticosteroid exposure. The exposure to topical and systemic corticosteroids and the inflammation itself might have a role in the pathogenesis of uveitic glaucoma and since the incidence of corticosteroid responsiveness in relatively higher in pediatric population, reducing steroid exposure might be beneficial. Ant TNFα agents are effective in both controlling the inflammation and decreasing the corticosteroid exposure.

Systemic conventional immunosuppressive treatment is effective in controlling inflammation and preserving visual acuity in JIA-associated uveitis. Biologic agents are also effective in reducing the corticosteroid exposure and improving the clinical outcomes.

## Data Availability

Data available on request from the authors. The data that support the findings of this study are available from the corresponding author, [D.B.], upon reasonable request.

## Declarations

### Human and animal participants

No animal studies are presented in this manuscript. The studies involving humans were approved by Ege University Ethics Committee for Medical Studies. The studies were conducted in accordance with the local legislation and institutional requirements. Written informed consent for participation in this study was provided by the participants’ legal guardians/next of kin. No potentially identifiable images or data are presented in this study.
